# From Synaptic Protein to Prion: The Long and Controversial Journey of α-Synuclein

**DOI:** 10.3389/fnsyn.2020.584536

**Published:** 2020-09-21

**Authors:** Antonio Heras-Garvin, Nadia Stefanova

**Affiliations:** Division of Neurobiology, Department of Neurology, Medical University of Innsbruck, Innsbruck, Austria

**Keywords:** α-synuclein, α-synucleinopathies, Parkinson’s disease, neurodegeneration, prion

## Abstract

Since its discovery 30 years ago, α-synuclein (α-syn) has been one of the most studied proteins in the field of neuroscience. Dozens of groups worldwide have tried to reveal not only its role in the CNS but also in other organs. α-syn has been linked to several processes essential in brain homeostasis such as neurotransmitter release, synaptic function, and plasticity. However, despite the efforts made in this direction, the main function of α-syn is still unknown. Moreover, α-syn became a protein of interest for neurologists and neuroscientists when mutations in its gene were found associated with Parkinson’s disease (PD) and even more when α-syn protein deposits were observed in the brain of PD, dementia with Lewy bodies (DLB), and multiple system atrophy (MSA) patients. At present, the abnormal accumulation of α-syn constitutes one of the pathological hallmarks of these disorders, also referred to as α-synucleinopathies, and it is used for post-mortem diagnostic criteria. Whether α-syn aggregation is cause or consequence of the pathogenic events underlying α-synucleinopathies remains unclear and under discussion. Recently, different *in vitro* and *in vivo* studies have shown the ability of pathogenic α-syn to spread between cells, not only within the CNS but also from peripheral locations such as the gut, salivary glands, and through the olfactory network into the CNS, inducing abnormal misfolding of endogenous α-syn and leading to neurodegeneration and motor and cognitive impairment in animal models. Thus, it has been suggested that α-syn should be considered a prion protein. Here we present an update of what we know about α-syn function, aggregation and spreading, and its role in neurodegeneration. We also discuss the rationale and findings supporting the hypothetical prion nature of α-syn, its weaknesses, and future perspectives for research and the development of disease-modifying therapies.

## Introduction

α-synuclein (α-syn) is one of the most abundant proteins in the nervous system, encoded by the *SNCA* gene. With a molecular mass of approximately 15 kDa, α-syn is composed of 140 amino acid residues (Lee and Trojanowski, 2006; Bendor et al., 2013). α-syn presents three domains: the C-terminal domain, the central domain, or “non-amyloid component” (NAC), which permits its oligomerization due to its hydrophobic composition, and the N-terminal domain, that forms an alpha helix allowing α-syn-lipid interaction (Lashuel et al., 2013). However, despite the efforts made since its discovery, its precise native structure is still unclear and under discussion. It has been proposed that, in physiological conditions, α-syn is mainly found in its monomeric form, either as a soluble intrinsically disordered protein in the cytoplasm of neurons (Binolfi et al., 2012; Fauvet et al., 2012; Theillet et al., 2016), or in an alpha-helical conformation (Bartels et al., 2011; Wang et al., 2011), or a combination of the two (Burré et al., 2013). Moreover, α-syn can undergo a variety of post-translational modifications (PTM; i.e., phosphorylation, glycation, glycosylation, acetylation) that may affect protein structure and function (Giasson et al., 2000; Theillet et al., 2016; Burré et al., 2018; Meade et al., 2019).

**Figure 1:**
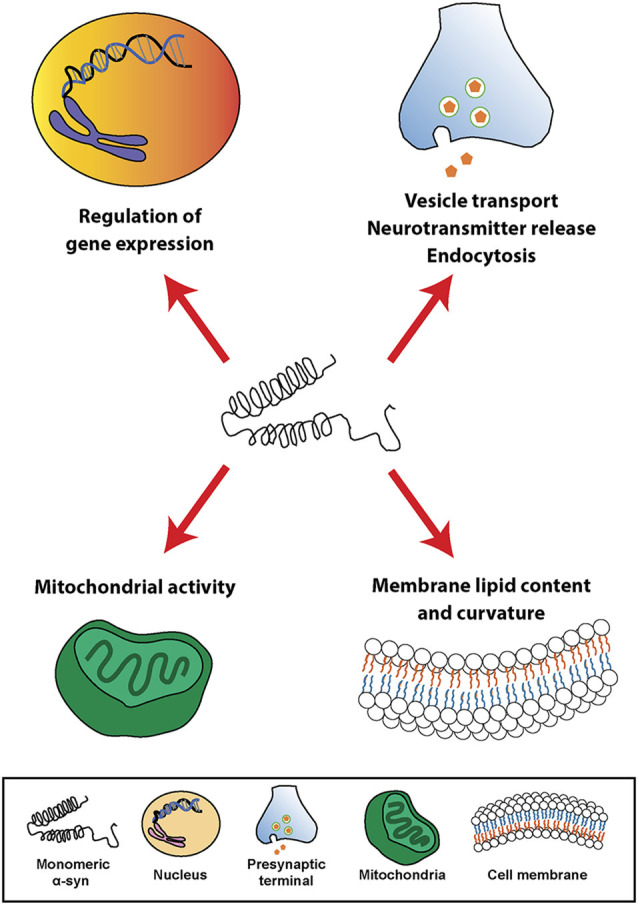
Physiological roles of α-synuclein (α-syn). Schematic overview including some of the cellular processes linked to α-syn in physiological conditions.

At the cellular level, α-syn can be found in different cellular organelles, including synaptic terminals and the nucleus of neurons (Maroteaux et al., 1988), mitochondria (Li et al., 2007; Devi et al., 2008; Parihar et al., 2008), endoplasmic reticulum (Hoozemans et al., 2007; Guardia-Laguarta et al., 2014), Golgi apparatus (Gosavi et al., 2002; Mori et al., 2002), and in the endolysosomal system (Lee et al., 2004). In this regard, since its discovery, α-syn has been associated or involved in different cellular processes, such as neurotransmission, calcium homeostasis, vesicle transport, mitochondrial function, and gene regulation (Figure 1). However, the physiological function of α-syn in those subcellular compartments is not fully understood. It has been shown that α-syn is involved in the regulation of the membrane lipid content and curvature, therefore playing a role in vesicle budding and trafficking (Chandra et al., 2003; Varkey et al., 2010). Moreover, α-syn plays a role in SNARE complex assembly at presynaptic terminals (Burré et al., 2010). Studies based on *in situ* proximity ligation assay (PLA) in neurons have demonstrated proximity between α-syn and SNARE proteins in cell bodies and neurites, including VAMP-2, SNAP-25, and syntaxin-1 (Almandoz-Gil et al., 2018). α-syn is also involved in clathrin-mediated endocytosis (Ben Gedalya et al., 2009) and in dopamine content and metabolism (Sidhu et al., 2004; Yu et al., 2005; Al-Wandi et al., 2010). In association with its nuclear localization, α-syn functions as a regulator of gene expression through its direct interaction with DNA (Pinho et al., 2019), chromatin (Vasquez et al., 2017), and transcription factors involved in development, mitochondrial homeostasis and metabolism of energy (Zheng et al., 2010; Decressac et al., 2012; Eschbach et al., 2015; Davidi et al., 2020), as well as through epigenetic mechanism including DNA methylation or histone acetylation (Surguchev and Surguchov, 2017). Furthermore, α-syn directly controls mitochondrial activity and function by modulating membrane potential, calcium homeostasis, cytochrome release, ATP production, and mitochondrial fusion and fission (Vicario et al., 2018). Interestingly, α-syn is also present in blood cells including red blood cells, peripheral blood mononuclear cells, platelets, and in the plasma. However, the role of α-syn in those cells is unclear (Barbour et al., 2008).

The functions and the structure of α-syn have being intensively investigated during the last decades, leading to a better knowledge of the protein and its association with crucial pathways on different cell types in the central nervous system (CNS), however α-syn research remains a priority not only for neuroscientist, but also for clinicians and pharmaceutical companies. The fact that α-syn aggregation and accumulation constitute the main pathological hallmark of a group of neurodegenerative disorders, referred to as α-synucleinopathies, and that mutations of its gene (*SNCA*) are associated with familiar forms of these disorders, has led α-syn to become one of the “most wanted” proteins in the biomedical field. Pre-clinical studies *in vitro* and *in vivo* have shown than α-syn oligomeric species and aggregates induce the impairment of different processes essential for cellular homeostasis, not only in neurons, but also in other cell types. Moreover, inoculation of α-syn oligomers, fibrils or aggregates in animal models, demonstrated the capability of pathogenic forms of α-syn to spread in a prion-like manner, inducing in some cases changes that replicated human pathology, including the clinical presentation. As a result of those observations, some authors have proposed that α-syn should be considered a prion protein. In this review, we discuss the role of α-syn in neurodegeneration, the different findings supporting the prion nature of α-syn, and its consequences for future therapeutic strategies.

## α-Syn and Neurodegeneration: α-Synucleionopathies

The abnormal aggregation and accumulation of α-syn constitute the main pathological hallmark of Parkinson’s disease (PD), multiple system atrophy (MSA), and dementia with Lewy bodies (DLB; Spillantini et al., 1997, 1998; Goedert and Spillantini, 1998). These three disorders are also known as α-synucleinopathies. Recently, pure autonomic failure (PAF) and REM sleep behavior disorder (RBD) have been suggested to represent prodromal α-synucleinopathies commonly preceding PD or MSA and showing α-syn aggregation in the peripheral and autonomic nervous system (Arai et al., 2000; Kaufmann et al., 2001, 2004; Iranzo et al., 2013; Barber et al., 2017). Polymeropoulos et al. (1996, 1997) first described the association between α-syn and PD, when they found that individuals with familiar forms of this disorder presented mutations in the SNCA gene (Polymeropoulos et al., 1996, 1997). Soon afterwards Spillantini et al. (1997, 1998) showed that the abnormal protein aggregates observed in α-synucleinopathies were strongly immunoreactive for α-syn (Spillantini et al., 1997, 1998; Goedert and Spillantini, 1998). Moreover, some *SNCA* gene allelic variants (i.e., SNCA locus triplication) were sufficient to develop a severe early onset PD and DLB (Singleton et al., 2003; Orme et al., 2018; Zafar et al., 2018). Since then, multiple *in vitro* and *in vivo* studies have tried to decipher the mechanisms underlying α-syn misfolding and aggregation, the following cellular and physiological consequences, and their influence in the human pathology.

α-synucleinopathies present a preference for affecting motor, cognitive and autonomic areas, leading to motor dysfunction, cognitive impairment and/or autonomic failure (Marti et al., 2003). Phenotypic diversity, symptom severity and disease duration varies among these disorders based on the affectation of different neuroanatomical regions, selective vulnerability of different cell types and the extent of the pathology (Marti et al., 2003). Pathologically, PD and DLB are characterized by the abnormal accumulation of α-syn in the cytoplasm of neurons, in the so-called Lewy bodies (LBs; Spillantini et al., 1998). Associated with the presence of LBs in cortical and subcortical areas, post-mortem evaluation of PD and DLB brains show a preferential loss of dopaminergic neurons in the substantia nigra pars compacta (SNpc), neuronal loss in the locus coeruleus (LC) and cortical atrophy (Dickson et al., 2009; Cheng et al., 2010; Jellinger, 2018). In addition to that, other neuronal populations in the brainstem accumulate LBs and degenerate, probably leading to many of the secondary clinical features of PD and DLB, such as autonomic dysfunction or sleep disturbances (Coon et al., 2018; Outeiro et al., 2019). In PAF, typical LBs are present in sympathetic ganglia, distal sympathetic nerves, substantia nigra, and locus ceruleus. In correlation with the presence of LBs, PAF patients present a significant unmyelinated nerve fiber density compared to healthy controls and neuronal degeneration in preganglionic sympathetic intermediolateral column and paravertebral ganglia (Kaufmann et al., 2001; Thaisetthawatkul, 2016). In MSA, however, α-syn mainly (but not exclusively) accumulates throughout the neocortex, hippocampus, brainstem, spinal cord, and dorsal root ganglia in the cytoplasm of oligodendrocytes, in the so-called glial cytoplasmic inclusions (GCIs), associating with striatonigral degeneration (SND), olivopontocerebellar atrophy and cell loss in LC and brainstem nuclei, leading to parkinsonism, cerebellar symptoms and autonomic dysfunction (Fanciulli and Wenning, 2015).

**Figure 2:**
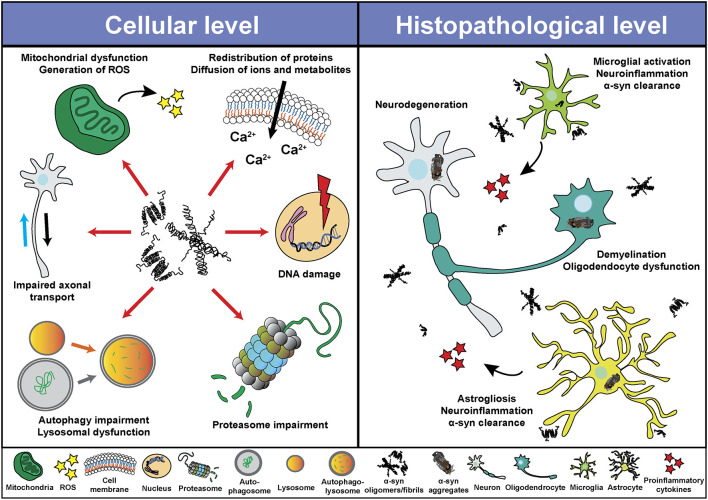
Pathological roles of α-syn. Schematic overview including some of the cellular processes (left) and histopathological events (right) associated with the presence of pathogenic α-syn species.

Despite the different clinical features of α-synucleinopathies, these disorders share characteristics such as neurodegeneration, neuroinflammation and demyelination, among others. Based on *in vitro* and *in vivo* studies, α-syn seems to play a central role in the pathological changes through a direct interaction with components of cellular pathways essential for cell homeostasis (Figure 2). Thus, pathological α-syn is able to bind and inhibit lysosomal function (Mazzulli et al., 2016; Flavin et al., 2017), proteasomal activity (Stefanis et al., 2001; Tanaka et al., 2001; Petrucelli et al., 2002; Snyder et al., 2003; Lindersson et al., 2004; Emmanouilidou et al., 2010), to impair axonal transport (Volpicelli-Daley, 2017), and to induce Ca^2+^ dyshomeostasis (Danzer et al., 2007; Chen Y. et al., 2015; Angelova et al., 2016) and mitochondrial dysfunction (Di Maio et al., 2016; Ganjam et al., 2019; Wang et al., 2019; Park et al., 2020). Latter promotes the generation of reactive oxygen species (ROS), and therefore oxidative stress, which may lead to mitochondrial damage, the release of cytochrome c to the cytoplasm, and cell death (Hsu et al., 2000; Smith et al., 2005; Parihar et al., 2008; Reeve et al., 2015; Tapias et al., 2017; Ludtmann et al., 2018). Associated with its nuclear localization or its capacity to promote oxidative stress, recent studies suggested an involvement of α-syn in DNA damage (Paiva et al., 2017; Vasquez et al., 2017; Milanese et al., 2018; Schaser et al., 2019). Pathological α-syn also promotes pathogenic redistribution of membrane proteins (Shrivastava et al., 2015, 2020) and interferes with the normal function of cell membranes, facilitating diffusion of molecules and ions, specially Ca^2+^, and inducing cell damage (Tosatto et al., 2012; Tsigelny et al., 2012; Angelova et al., 2016; Fusco et al., 2017; Dong et al., 2018). The impairment of all these cellular processes may be especially significant in dopaminergic neurons, since dopamine promotes the formation of pathological α-syn species (Cappai et al., 2005; Lee et al., 2011; Mor et al., 2017). Thus, a multi-hit model has been proposed, in which the synergistic interaction between α-syn, dopamine and Ca^2+^ in neurons may underlie neuronal loss in SNpc and LC (Post et al., 2018). Neurodegeneration in the LC may induce a direct loss of norepinephrine (NE), however, α-syn is also able to translocate to the nucleus of NE-producing cells and interfere with transcription of dopamine ß-hydroxylase, the final enzyme in NE biosynthesis, disrupting NE production and leading to a reduction of neurotrophic factor signaling, indirectly altering innate and adaptive immune response and worsening central and peripheral inflammation (Butkovich et al., 2018).

Different *in vivo* and *in vitro* studies have also shown the ability of pathological α-syn to induce synaptic dysfunction, both at the presynaptic and postsynaptic terminal (Ghiglieri et al., 2018). At presynaptic level, pathological α-syn seems to affect synaptic vesicle pool, trafficking and endocytosis by interfering with the exo-endocytotic cycling machinery, thus suggesting the possible association between α-syn pathology and impaired synaptic function even in the absence of cell death (Bridi and Hirth, 2018; Froula et al., 2018; Wu et al., 2019). At a postsynaptic level, *in vivo* studies have shown that pathological α-syn reduces striatal dopamine, NMDA receptor-mediated synaptic currents, impairs early memory and motor learning, and prevents long-term potentiation of cholinergic interneurons and spiny projection neurons in the striatum even at early stages of the disease (Tozzi et al., 2016; Phan et al., 2017; Giordano et al., 2018; Durante et al., 2019).

Pathological α-syn can also modulate neuroinflammation (Figure 2) directly through different mechanism including the activation of microglial cells (Austin et al., 2006; Su et al., 2008; Theodore et al., 2008; Fellner et al., 2013; Choi et al., 2020), which induces a robust pro-inflammatory response (Kim et al., 2013; Sarkar et al., 2020) leading to increased α-syn aggregation and potentiating neurodegeneration (Zhang et al., 2005; Stefanova et al., 2007). In this regard, microgliosis has been shown in post-mortem studies and in PET imaging analysis of patients diagnosed with α-synucleinopathies (McGeer et al., 1988a; Gerhard et al., 2003, 2006; Doorn et al., 2014; Stokholm et al., 2017; Surendranathan et al., 2018). In addition, microgliosis paralleled system degeneration in patients and was associated with α-syn inclusions (Imamura et al., 2003; Ishizawa et al., 2004, 2008; Croisier et al., 2005; Kubler et al., 2019). A recent study with PD patients engrafted with foetal embryonic dopaminergic neurons showed microglia activation in all grafts before α-syn aggregates were detected (Olanow et al., 2019). The authors speculated that microglial activation could have been initiated by the presence of endogenous pathological α-syn in patients, by an immune reaction due to the implantation of foreign tissue or by the surgical procedure itself (Olanow et al., 2019). Moreover, we recently showed that targeting α-syn oligomerization in an animal model for MSA led to a decrease of α-syn aggregates which significantly correlated with neuroprotection and a reduction in microglial activation (Heras-Garvin et al., 2019).

Similar to microglial cells, pathological α-syn also induces activation of astrocytes (Figure 2), aggravating neuroinflammation (Lee et al., 2010; Fellner et al., 2013; Radford et al., 2015). Astrogliosis has also been described in α-synucleinopathies paralleling the neurodegenerative process (Ozawa et al., 2004; Miklossy et al., 2006; Radford et al., 2015). Moreover, α-syn inclusions are often found in the cytoplasm of astrocytes and may lead to astrocyte dysfunction (Song et al., 2009; Halliday and Stevens, 2011; Sorrentino et al., 2019). Even a role of the adaptive immune system in PD has been proposed. Post-mortem evaluation showed higher numbers of T cells in the ventral midbrain of PD brains and mouse models than in healthy controls, suggesting a targeted extravasation, and supporting a role for T cells in disease pathogenesis (McGeer et al., 1988b; Theodore et al., 2008; Brochard et al., 2009; Sommer et al., 2018). Recent studies showed the presence of α-syn-specific T cells in PD patients, and therefore an autoimmune response to α-syn (Sulzer et al., 2017; Garretti et al., 2019). However, at present, α-syn-specific T cells have only been detected in the periphery of PD patients. Whether these cells are able to infiltrate into the CNS and cause dopaminergic neuron death remains to be determined.

Although most research on the pathogenic role of α-syn have been focused in neurons, microglia and astrocytes, there have also been studies that evaluated the deleterious effect of pathogenic α-syn in oligodendrocytes and oligodendroglial precursor cells (OPCs), which are essential to understand the cellular and molecular consequences of α-syn accumulation in MSA (Figure 2). Over-expression of α-syn or the addition of exogenous pathological α-syn severely impaired myelin formation *in vitro* and *in vivo* (Ettle et al., 2016; Mandel et al., 2017; Mavroeidi et al., 2019), corroborating the demyelination observed in MSA brains (Song et al., 2007; Don et al., 2014; Ettle et al., 2016). This effect seems to be mediated by the interference of α-syn with the expression of proteins associated with cholesterol and membrane biogenesis (Ettle et al., 2016; Kaji et al., 2018), and by the partial sequestration of the myelin basic protein (MBP) into GCIs in a process orchestrated by endogenous α-syn and the the oligodendroglial-specific phosphoprotein p25α (Mavroeidi et al., 2019). Moreover, α-syn impairs OPC maturation *in vitro* (Ettle et al., 2014) and in animal models of MSA, where an age-dependent increase of dividing OPCs within the striatum was observed (May et al., 2014). Latter was confirmed after postmortem analysis in MSA patients, revealing α-syn within OPCs and an increased number of striatal OPCs compared to healthy controls (May et al., 2014). α-syn also induces a distinct gene expression profile in oligodendrocytes, including upregulation of cytokines important for myeloid cell attraction and proliferation (Schafferer et al., 2016; Hoffmann et al., 2019), which constitutes an early crosstalk between neuroinflammation and α-syn-mediated oligodendrocyte dysfunction.

Overall, multiple cell populations and pathways can be affected by the abnormal misfolding, aggregation and accumulation of α-syn. Determining which events are primary or secondary in the pathogenesis of α-synucleinopathies will be essential in order to define potential strategies to prevent cell damage and, finally, neurodegeneration.

## Aggregation and Spreading of α-Syn

**Figure 3:**
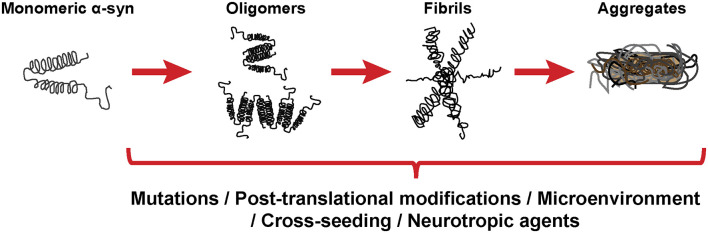
α-synuclein aggregation process. Schematic overview of the hypothetical aggregation process of monomeric α-syn and the different events that can induce its misfolding and promote aggregation.

As stated before, the normal conformation of α-syn is either a disordered monomer, alpha-helical, or a combination of the two. But, under pathological conditions, monomers can convert to β-sheets by recruiting additional monomers, aggregating and giving rise to oligomers that, eventually, form protofilaments and amyloid fibrils (Figure 3), which can adopt different morphologies such as spherical, chain-like, annular (pore-like structure), and tubular (Cremades and Dobson, 2018). To define the process through which α-syn oligomers give rise to pathological inclusions is challenging due to their transient nature and their ability to rapidly recruit monomeric α-syn to form fibrils. Nevertheless, the recent description that α-syn oligomers can be kinetically trapped made possible to compare the contribution of oligomers and fibrils to different α-synucleinopathy phenotypes (Chen S. W. et al., 2015; Iljina et al., 2016; De Oliveira and Silva, 2019; Froula et al., 2019). It is believed that the formation of α-syn fibrils results from interactions between monomers and oligomers that are thermodynamically favorable and stabilizing (Alam et al., 2019). Despite several studies have associated the presence of α-syn oligomers and fibrillar species with cytotoxicity and neurodegeneration, thus supporting the idea that these α-syn forms are the responsible for the neurodegenerative process observed in α-synucleinoptahies (Alam et al., 2019), a recent publication by Mahul-Mellier et al. (2020) proposed that the process of LB formation, rather than simply fibril formation, is one of the major drivers of neurodegeneration through disruption of cellular functions, inducing mitochondria damage and deficits, and synaptic dysfunction. Therefore, which α-syn species or processes associated with α-syn aggregation and accumulation are the most toxic is still a matter of debate.

Mutations in the *SNCA* gene associated with familiar forms of α-synucleinopathies, such as A30P or A53T, are more prone to aggregate and form fibrillar α-syn oligomers (Lashuel et al., 2002; Bengoa-Vergniory et al., 2017). Different events promote α-syn aggregation (Figure 3), including low pH (Buell et al., 2014). Indeed, neutral pH inhibits the growth of α-syn fibrils, while acidic pH increases it (Grey et al., 2011). Other micro-environmental factors affecting α-syn aggregation include metal ions (Uversky et al., 2001), polyamines (Krasnoslobodtsev et al., 2012; Holmes et al., 2013), proteoglycans (Lashuel et al., 2013), nucleic acids (Hegde and Rao, 2007), poly-ADP ribose (Kam et al., 2018) and fatty acids (Fecchio et al., 2018). α-syn PTM play also a role in its aggregation process. Thus, glycation of α-syn potentiate toxicity and aggregation (Vicente Miranda et al., 2017). Phosphorylation at various sites contributes to α-syn aggregation (Zhang et al., 2019). In particular, phosphorylation at residue Ser129 constitutes one of the most studied α-syn PTM, however, there is contradictory data about its role in pathology, as it was shown to be both protective and toxic (Arawaka et al., 2017; Zhang et al., 2019). In physiological conditions, α-syn is constitutively acetylated in its N-terminal, which seems to make α-syn more resistant to oligomerization and aggregation (Gonzalez et al., 2019). C-terminal truncation of α-syn has been associated with an increase in self-assembly properties and constitutes 10–25% of the LBs (Li et al., 2005; Wang et al., 2016). As a result of oxidative stress, the generation of nitric oxide induces nitration of tyrosine residues of α-syn, promoting aggregation (Giasson et al., 2000). SUMOylation inhibits ubiquitin-mediated degradation of α-syn, which leads to an increase of α-syn concentration and aggregation (Rott et al., 2017). Lastly, the aggregation process of α-syn seems to be affected by the presence of other proteins. Although homotypic seeding is the preferred form of aggregation, α-syn can bind other natively unfolded proteins such as tau, PrP^C^ or Aβ, in a process termed heterotypic cross-seeding, which accelerate aggregation and neuropathology (Clinton et al., 2010; Ono et al., 2012; Guo et al., 2013; Katorcha et al., 2017; Bassil et al., 2020). However, despite our knowledge about the different processes that may be involved in its abnormal aggregation, the initial event that triggers α-syn oligomer formation and accumulation remains unknown.

In 2003, after post-mortem evaluation of PD brains, Braak et al. (2003a) staged the pathology of this disorder. In that study, they defined the earliest phases of the disease, characterized by the presence of α-syn pathology in the anterior olfactory nucleus and in the dorsal motor nucleus of the vagal in the absence of motor symptoms. Based on those findings, Braak et al. (2003b) developed a theory in which PD might be initiated when a neurotropic agent enters the body by nasal route, through the olfactory epithelium to the olfactory bulb, or by gastric route, infiltrating the gut to the submucous plexus and traveling along preganglionic parasympathetic fibers to the dorsal motor nucleus of the vagus nerve. The presence of LBs in the enteric and peripheral nervous systems supported their claim (Braak et al., 2006, 2007; Shannon et al., 2012). The LB pathology would then travel through the CNS. The authors proposed that PD could be divided into six different stages, each one defined by abnormal pathology in particular neurological structures and specific symptom severity. Thus, early stages would be characterized by non-motor symptoms, including hyposmia and autonomic dysfunction, mid-stages would be characterized by the presence of motor symptoms and later stages by the presence of cognitive impairment (Braak et al., 2004). In their theory, the authors proposed as the possible neurotropic agent a virus or a pathogen composed of fragments of misfolded α-syn that could possess unconventional prion-like properties (Braak et al., 2003b). Over the last decade, different studies have shown that α-syn is in fact not only able to aggregate, but also to spread and propagate the synucleinopathy in a prion-like fashion. This new hypothesis and line of research was initially inspired by the observation that grafts of embryonic dopamine neurons into the brains of human PD patients developed α-syn pathology (Kordower et al., 2008; Li et al., 2008). The authors hypothesized that pathological α-syn from the host was transferred into the young grafted neurons in a prion-like manner (Brundin et al., 2008). Since then, different groups have proven that α-syn is able to be taken up by cells, including dopaminergic neurons (Luk et al., 2009; Volpicelli-Daley et al., 2011), be transmitted from neuron to neuron (Desplats et al., 2009; Freundt et al., 2012), from neurons to oligodendrocytes (Reyes et al., 2014), and to induce the formation of aggregates that share some of the LB features, i.e., a high degree of phosphorylation and polyubiquitination (Volpicelli-Daley et al., 2011). An early work demonstrated the presence of α-syn in plasma and cerebrospinal fluid of PD patients, thus supporting the hypothesis that α-syn may enter cells from the extracellular space (El-Agnaf et al., 2003). Moreover, the inoculation of recombinant α-syn preformed-fibrils (PFFs) or patient-derived pathological α-syn in wildtype and transgenic animals resulted in the development of widespread synucleinopathy throughout synaptically connected networks, neurodegeneration and behavioral deficits, following in some cases a similar pattern to the one define in Braak’s hypothesis (Luk et al., 2012a,b; Masuda-Suzukake et al., 2013; Ulusoy et al., 2013; Holmqvist et al., 2014; Recasens et al., 2014; Paumier et al., 2015; Abdelmotilib et al., 2017; Manfredsson et al., 2018; Rey et al., 2018; Chu et al., 2019; Kim S. et al., 2019; Challis et al., 2020). Additional studies demonstrated that the spreading of α-syn pathology depended on the misfolding and recruitment of endogenous α-syn (Volpicelli-Daley et al., 2011; Kaji et al., 2018; Kim S. et al., 2019; Wu et al., 2019). Furthermore, a recent publication suggested that the inoculation site may determine the synucleinopathy, since autonomic ganglionic injection of α-syn fibrils resulted in a model of PAF (Wang et al., 2020). However, the mechanism involved in α-syn internalization remains unclear. In the last years, several membrane protein receptors have been proposed to play an important role in α-syn uptake, including lymphocyte-activation gene 3 (Mao et al., 2016), Rab proteins (Masaracchia et al., 2018) and flotillin-1 and dopamine transporter (DAT; Kobayashi et al., 2019).

In addition, recent publications have demonstrated the existence of different strains of pathological α-syn which cause distinct synucleinopathies, targeting distinct brain regions and cell types (Bousset et al., 2013; Peelaerts et al., 2015; Rey et al., 2019; Lau et al., 2020). In particular, MSA and PD strains seem to have different structural and seed properties that may be used as differential diagnostic criteria and that could explain the more aggressive and rapid progression of MSA compared to other synucleinopathies (Peng et al., 2018b; Candelise et al., 2019; Yamasaki et al., 2019; Schweighauser et al., 2020; Shahnawaz et al., 2020; Van Der Perren et al., 2020). It is not yet clear how the different α-syn strains are generated, but the cellular micro-environment may play an important role (Candelise et al., 2020). Thus, a recent publication by the group of Virginia Lee showed that the α-syn extracted from GCIs presents a different proteolytic profile and more potent biological activity than the α-syn from LBs and that the specific cellular milieu of oligodendrocytes is responsible for the transformation of misfolded α-syn into the MSA strain (Peng et al., 2018a).

The identification of the mechanisms associated or responsible for α-syn uptake, processing, aggregation and release, and the contribution of different α-syn strains to inclusion formation and to the impairment of cellular pathways will be essential to completely understand the pathogenic process underlying α-synucleinopathies, to identify novel targets for disease modification, the development of new therapeutic strategies and to generate possible biomarkers which would allow the early diagnosis of these disorders.

## α-Syn, a Prion Protein?

**Figure 4:**
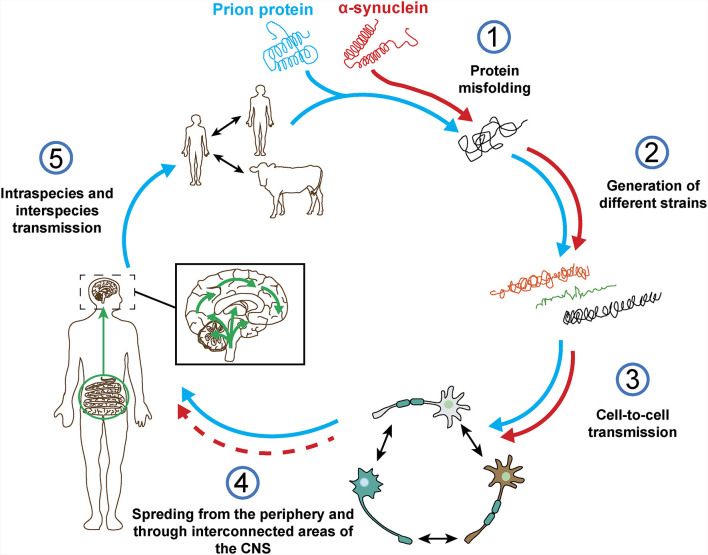
Prion protein vs. α-synuclein: current knowledge. Both proteins can undergo misfolding (1) which leads to the formation of different pathogenic strains (2). The pathogenic forms of both proteins can be transferred cell-to-cell (3) and spread from the periphery into the CNS *in vivo* (4). However, based on post-mortem data from α-synucleinopathy patients and in contrast to the prion protein, it is not clear how and where does α-synuclein pathology start and if the spreading observed in *in vivo* studies really happens in humans (4). Finally, unlike prion protein, no evidence of intraspecies or interpecies transmission of α-syn has ever been reported (5).

The term prion was first described by Stanley Prusiner as a “small proteinaceous infectious particle which is resistant to inactivation by most procedures that modify nucleic acids” and resulted from the blend of “protein” and “infection” (Prusiner, 1982). Therefore, prions should constitute the underlying cause of an infectious disease that can be transmitted among individuals from the same or different species, and not a secondary consequence of the disease (Prusiner, 1998). In this regard, based on *in vivo* and *in vitro* studies, α-syn seems to exhibit some of the properties of prion proteins (Figure 4), such as the ability to undergo misfolding and to self-propagate, inducing aggregation of endogenous α-syn (Hijaz and Volpicelli-Daley, 2020), to exist as distinct strains (Bousset et al., 2013; Peelaerts et al., 2015; Candelise et al., 2019; Yamasaki et al., 2019; Lau et al., 2020; Schweighauser et al., 2020; Shahnawaz et al., 2020) and to be resistant to inactivation by formaldehyde (Schweighauser et al., 2015; Woerman et al., 2018). As discussed before, the fact that α-syn aggregates were found in human brain regions affected by the disease and that some studies, like the one conducted by Braak et al. (2004) showed the presence of inclusions in peripheral organs and described an association between disease stage and Lewy pathology, led the authors to propose that α-synucleinopathies could initiate in the gut or the olfactory bulb and slowly spread to the CNS (Beach et al., 2009). This evidence and the data demonstrating the ability of α-syn to propagate from the gut and other peripheral organs in animal models (Kim S. et al., 2019; Lohmann et al., 2019) as well as the finding of LBs in grafted dopaminergic neurons in PD patients (Kordower et al., 2008; Li et al., 2008) supported the prion nature of α-syn.

However, there is evidence that challenges some of these observations and the prion hypothesis for α-syn (Figure 4). On one hand, subsequent post-mortem studies showed that only half of PD cases present a pattern of Lewy pathology consistent with Braak’s staging and that an important number do not even follow it (Kalaitzakis et al., 2008; Jellinger, 2009; Halliday et al., 2012). In many cases, the distribution of α-syn inclusions is sparse and discrete, even at late stages of the disease (Beach et al., 2009; Dijkstra et al., 2014), and some patients do not show aggregates at all (Berg et al., 2014). Moreover, no correlation has been observed between Braak’s staging of Lewy pathology and severity of clinical progression (Jellinger, 2009). In addition, cells within the same region are not equally affected by Lewy pathology, which is only observed in a small percentage of neurons, mainly affecting catecholaminergic and cholinergic but not GABAergic neurons. Furthermore, the strength of connectivity between brain areas does not correlate with Lewy pathology patterns (Surmeier et al., 2017) and, in MSA, the trans-synaptic propagation of α-syn pathology proposed for PD cannot explain the spatial distribution of GCIs (Armstrong et al., 2004; Dhillon et al., 2019; Kaji et al., 2020).

The ability of α-syn to propagate and generate aggregates in humans is also a matter of debate since additional transplantation studies did not show α-syn accumulation in the engrafted dopaminergic cells (Mendez et al., 2008; Hallett et al., 2014). Interestingly, the main difference between these studies and the ones showing Lewy pathology was the use of dissociated neurons instead of cellular aggregates or tissue pieces. Moreover, independently of the absence or presence of α-syn aggregates, transplanted neurons generally survive and remain healthy and functional for decades (Li et al., 2016). The appearance of pathology in the engrafted cells has been, however, associated with an extensive microglial activation when cellular aggregates or tissue pieces are used (Kordower et al., 2008; Li et al., 2008). This effect was not observed when dissociated neurons were transplanted (Mendez et al., 2008). Nevertheless, it is still unclear from where was the pathology observed in transplanted cells coming from, and why the number of neurons affected did not change with time (Kordower et al., 2008; Li et al., 2008, 2016). It has been proposed that the graft’s micro-environment together with the already stressed environment from the host may trigger the pathology in those cells (Surmeier et al., 2017). In fact, post-mortem studies have shown that brain regions presenting α-syn inclusions do not necessarily develop neuronal loss, in contrast, neurodegeneration sometimes occurs in areas with no α-syn pathology or before it appears (Leak et al., 2019). Furthermore, *in vitro* studies have shown that mutations in α-syn, such as A53E and G51D, which are associated with familiar forms of PD and MSA, attenuate aggregation and delay the pathology (Fares et al., 2014; Ghosh et al., 2014). Interestingly, different neuropathological reports have shown an absence of Lewy pathology in most patients with familiar forms of parkin-related PD and in a proportion of PD patients with mutations in the *LRRK2* gene, which are respectively the most common cause of recessive and dominant forms of PD, and a small proportion of sporadic PD cases (Kalia and Lang, 2015; Tolosa et al., 2020).

Bonafide prions can spread from extraneural sites into the CNS and the molecular processes underlying their spreading have been evaluated in detail (Kara et al., 2018). Regarding α-syn, recent studies have demonstrated the presence of inclusions in peripheral sites, including cells of the enteric nervous system, nerve cells and fibers in the skin and submandibular salivary glands of α-synucleinopathies patients (Beach et al., 2010, 2016; Shannon et al., 2012; Kim J. Y. et al., 2019). Therefore, these data support the hypothesis that α-syn pathology may start in the periphery and ascend towards the CNS in a similar way to prions. In addition, it was reported that vagotomy reduces the risk of developing PD (Svensson et al., 2015), that may be explained by a disruption of α-syn spreading from the periphery to the brain. However, these observations were not replicated in a different study with the same cohort but extended follow-up (Tysnes et al., 2015). Therefore, the debate on the periphery as the possible initiation site of α-syn pathology remains unsolved, especially considering that: (i) α-syn is mainly expressed in the brain (Lashuel et al., 2013); (ii) some peripheral regions with α-syn pathology do not present a clear exposure route, such as the cardiac sympathetic nerve (Orimo et al., 2008); (iii) no cases have been found in post-mortem whole body studies with α-syn inclusions in the periphery without the brain being affected (Lionnet et al., 2018); and (iv) peripheral α-syn aggregates from PD patients show lack of pathogenic potential (Recasens et al., 2018; Figure 4).

Other exogenous and endogenous factors may trigger the pathological changes underlying α-synucleinopathies and α-syn aggregation (Figure 3). Thus, gut dysbiosis and microbiota have been associated with PD (Fitzgerald et al., 2019). Moreover, recent studies demonstrated that amyloid proteins produced by gut bacteria can induce α-syn aggregation and seeding (Sampson et al., 2016, 2020), and that viral infection disrupts cellular proteostasis and causes α-syn aggregation (Marreiros et al., 2020). Several authors have therefore suggested that neurodegeneration and the neurological deficits observed in α-synucleinopathies may be triggered by factors other than α-syn, like inflammation or local micro environmental conditions (Engelender and Isacson, 2017) and that selective vulnerability of neuronal subpopulations may better explain the generation of α-syn inclusions throughout disease progression, rather than prion-like transmission (Surmeier et al., 2017). In this regard, a protective role of α-syn aggregates has been suggested, where they may be a consequence of the disease process rather than the cause (Olanow et al., 2004; Sian-Hulsmann et al., 2015). Based on these observations, and the fact that severity of clinical symptoms correlates with neurodegeneration rather than with α-syn inclusions in humans (Beach et al., 2009), the role of α-syn in these pathologies remains unclear.

It has to be mention that, to date, despite longitudinal studies with α-synucleinopathies patients have demonstrated clinical and biological changes throughout disease progression, they have not been able to show meaningful concordance between them, making difficult to properly define disease stages and, therefore, to evaluate the association between α-syn pathology and disease progression (Espay et al., 2020). In addition to that, the absence of significant correlation between α-syn pathology and cell loss in brain areas affected in these disorders could be due to a lack of sensitivity of current immunohistochemical methods. Further studies evaluating the presence of α-syn oligomers or early aggregation stages by novel techniques such as AS-PLA, RT-QuIC or PMCA may lead to a better understanding of the role of α-syn in α-synucleinopathies and clarify the possible pathological routes of α-syn propagation (Bengoa-Vergniory et al., 2017; Shahnawaz et al., 2020).

Finally, no evidences of α-syn transmission between humans has ever been reported, including iatrogenic (Irwin et al., 2013; Beekes et al., 2014), and there is no epidemiological data supporting this possibility for any α-synucleinopathy (Rajput et al., 2016; Figure 4). No evidences of transmission from animal to human have been found either (Wenning et al., 2018). Historically, prion infections have been a rarity whereas α-synucleinopathies present an important incidence that remains relatively stable over time in various ethnical populations (Pringsheim et al., 2014; Leak et al., 2019), however, *in vivo* studies show that the level of infectivity of α-syn aggregates per amount of protein might be orders of magnitude lower than that of prions (Watts, 2019).

Overall, despite α-syn shares several characteristics with prions, the absence of infectivity, the lack of human studies supporting the hypothesis and the existence of contradictory data must be taken in consideration. Thus, the term “prionoid” has been proposed to designate proteins that share propagative mechanisms with prions but may not be transmissible between individuals (Kara et al., 2018). At present, the most probable explanation regarding the start and progression of α-synucleionopathies would be that they are the consequence of a multifactorial process. This process would include the occurrence of exogenous and cellular micro-environmental changes in the affected regions that may trigger and modulate the pathology, and the presence of different α-syn strains that are able to spread through the organism in a cell type/susceptibility-dependent manner leading to different neuroanatomical spreading patterns.

## Futures Perspectives in α-Syn Research and Therapy

Although our knowledge about α-syn, its physiological functions, structure, properties and its pathological role in α-synucleinopathies have been extensively studied, there are still gaps that must be filled to completely understand the nature and the importance of this protein.

The characterization of α-syn interactions in the different subcellular compartments, not only in the CNS, but also in peripheral organs may be essential to fully understand its functions, the cellular and systemic changes that underlie α-synucleinopathies, and the possible deleterious consequences of therapeutic strategies targeting α-syn. The combination of novel super-resolution imaging, proteomics and computational techniques constitute a powerful approach to define α-syn interactome (Brás et al., 2020). A complete and detailed identification of all α-syn PTM, the different pathways involved in its clearance and the role of glial cells in this process represent other important targets for further studies (Lopes Da Fonseca et al., 2015; Gonzalez et al., 2019; Tremblay et al., 2019). Regarding the pathological role of α-syn, the absence of specific biomarkers and PET tracers constitute, at present, one of the most important disadvantages to identify its spatial and temporal contribution to disease progression in α-synucleinopathy patients. In addition, the biochemical composition and the molecular architecture of α-syn strains and inclusions and the cellular and environmental factors modulating their formation in the different disorders have not yet been completely revealed, mainly due to the lack of proper and highly sensitive protocols and tools. Elucidating the native conformation of α-syn and the different pathogenic strains by cryo-electron microscopy (Chen S. W. et al., 2015; Li et al., 2018; Guerrero-Ferreira et al., 2019), nuclear magnetic resonance (Galvagnion et al., 2019) or super-resolution imaging techniques (Nugent et al., 2017; Shahmoradian et al., 2019) will be essential to develop alternative drug-based disease modifying therapies. The establishment of brain banks of high-quality post-mortem brain tissue, CSF, plasma and fibroblast from clinically and pathologically well-defined patients and healthy controls will improve the potential of future studies. Moreover, the incorporation of induced-pluripotent stem cells (iPSCs) to those banks, to develop human patient-specific *in vitro* models of the disease, should be considered. Finally, further genetic and epigenetic studies will also provide important insights into the mechanism underlying α-synucleinopathies, the differences between them, as well as novel therapeutic targets.

Though the exact role of α-syn in the pathology has not yet been fully elucidated, an important number of preclinical studies and clinical trials have been focused on targeting directly α-syn and α-syn aggregation, or indirectly through the modulation of processes associated with its accumulation. The use of small molecules to target α-syn aggregation have shown positive results in preclinical models of α-synucleionopathies by reducing aggregation and accumulation of α-syn inclusions, neurodegeneration and motor symptoms (Finkelstein et al., 2017; Price et al., 2018; Heras-Garvin et al., 2019; Wegrzynowicz et al., 2019). Antibiotics such as rifampicin also reduces α-syn aggregation and fibrillization (Li et al., 2004) and showed positive results in animal models (Ubhi et al., 2008). Different preclinical studies have demonstrated that α-syn immunotherapy enhances α-syn clearance, prevents spreading and neuronal loss and demyelination and improves motor function in animal models (Masliah et al., 2011; Mandler et al., 2014, 2015). Enhancing α-syn degradation by modulating cellular pathways and the activation of glial cell types associated with α-syn clearance constitute another promising strategy (Malagelada et al., 2010; Gao et al., 2019; Perrino et al., 2019). Stimulation of microglial-dependent clearance of α-syn reduces the number of inclusions, ameliorates motor symptoms and shows a neuroprotective effect in *in vivo* models of MSA and PD (Park et al., 2016; Venezia et al., 2017; Choi et al., 2020). Intriguingly, microglial activation and neuroinflammation can also promote α-syn aggregation and induce neurodegeneration, accelerating disease progression (Lema Tome et al., 2013), therefore, reduction of microglial activation have also been used for disease modification (Liu et al., 2019). Recently, the use of antisense nucleotides (ASO) to selectively inhibit proteins associated with α-syn pathology in specific brain regions or cell types has been proposed as an alternative approach to reduce α-syn accumulation in PD models (Zhao et al., 2017; Alarcón-Arís et al., 2018). This is particularly important since altering or removing α-syn gene expression in previous studies has shown profound and deleterious intracellular and developmental effects that in some cases could induce neuronal loss (Perez and Hastings, 2004; Benskey et al., 2016).

Most of these therapeutic strategies are currently under evaluation in human clinical trials (Heras-Garvin and Stefanova, 2020). Unfortunately, some of them have not shown ability to slow or halt disease progression (Heras-Garvin and Stefanova, 2020). The distinct efficacies observed between preclinical studies, based on *in vitro* and *in vivo* models, and the data from human studies could be explained by the neurobiological differences among species, but may be a consequence of current limitations to recruit patients at early stages of the disease, when significant neurodegeneration and motor symptoms are not yet present, to misdiagnosis and to the absence of specific biomarkers for α-syn.

## Conclusions

It has been a long journey since α-syn was discovered 30 years ago. Many *in vitro* and *in vivo* studies have contributed to a better understanding of this versatile and multifaceted protein, its functions and its potential role in neurodegeneration. Moreover, the analysis of human material has indeed supported the idea that α-syn may contribute in some extent to the pathogenic events underlying the α-synucleionopathies. However, whether α-syn is the cause or main force driving these disorders or a mere secondary event is still a matter of debate. The use of novel highly sensitive tools and techniques in future studies, combined with the recruitment and collection of human material from well-characterized and documented cohorts, will definitely help us to finally define the pathogenic role of α-syn and to improve the development of proper therapeutic strategies.

## Author Contributions

AH-G: conception and design, drafting and revising the manuscript, and final approval of the submission. NS: conception and design, revising the manuscript, and final approval of the submission. All authors contributed to the article and approved the submitted version.

## Conflict of Interest

The authors declare that the research was conducted in the absence of any commercial or financial relationships that could be construed as a potential conflict of interest.
